# Regulator of G Protein Signaling 1 Suppresses CXCL12-Mediated Migration and AKT Activation in RPMI 8226 Human Plasmacytoma Cells and Plasmablasts

**DOI:** 10.1371/journal.pone.0124793

**Published:** 2015-04-21

**Authors:** Hyo-Kyung Pak, Minchan Gil, Yoonkyung Lee, Hyunji Lee, A-Neum Lee, Jin Roh, Chan-Sik Park

**Affiliations:** 1 Cell Dysfunction Research Center, University of Ulsan College of Medicine, Seoul, Korea; 2 Institute for Life Sciences, Asan Medical Center, University of Ulsan College of Medicine, Seoul, Korea; 3 Department of Pathology, Asan Medical Center, University of Ulsan College of Medicine, Seoul, Korea; Imperial College London, UNITED KINGDOM

## Abstract

Migration of plasma cells to the bone marrow is critical factor to humoral immunity and controlled by chemokines. Regulator of G protein signaling 1 (RGS1) is a GTPase-activating protein that controls various crucial functions such as migration. Here, we show that RGS1 controls the chemotactic migration of RPMI 8226 human plasmacytoma cells and human plasmablasts. LPS strongly increased RGS1 expression and retarded the migration of RPMI 8226 cells by suppressing CXCL12-mediated AKT activation. RGS1 knockdown by siRNA abolished the retardation of migration and AKT suppression by LPS. RGS1-dependent regulation of migration via AKT is also observed in cultured plasmablasts. We propose novel functions of RGS1 that suppress AKT activation and the migration of RPMI 8226 cells and plasmablasts in CXCL12-mediated chemotaxis.

## Introduction

Migration of plasma cells to bone marrow determines the function of plasma cells which is crucial for humoral immunity [1,2]. Homing of plasma cell neoplasm, plasmacytoma, to bone marrow is also critical to their survival and drug resistance [3,4]. Migration of plasma cells and plasmacytomas are controlled by chemokine-chemokine receptor signaling, most importantly CXCR4-CXCL12 axis. Chemokine receptors mostly consist of heptahelical G protein-coupled receptors (GPCR) that link to downstream signaling pathways by activating heterotrimeric G proteins [5]. G proteins consist of 3 subunits: α, β, and γ [6]. Upon GPCR activation, the α subunit releases guanosine diphosphate, binds guanosine triphosphate (GTP), and dissociates from the βγ subunit. This reaction activates downstream molecules, such as mitogen-activated protein kinases (MAPK), including p44/42 MAPK (also known as the extracellular signal-regulated kinase [ERK]), p38 MAPK, JAK/STAT, and AKT [7,8,9]. The PI3K/AKT signaling pathway plays a critical role in mediating survival signals. Recent studies report that this signaling axis also regulates migratory processes. PI3K/AKT controls the velocity of mesodermal cell migration and leads to actin polymerization [10].

Regulator of G protein signaling (RGS) proteins also regulate GPCR signaling [7]. There are more than 20 distinct RGS proteins, but all share an RGS box that consists of approximately 120 amino acids that bind to the α subunit of heterotrimeric G proteins and act as GTPase-activating proteins that accelerate GTP hydrolysis and signal termination [11]. Moreover, RGS proteins can associate with the β subunits through G protein γ-subunit-like domains and interfere with the actions of the β subunit in effector systems [12]. The inhibitory effects of lymphocyte migration by the RGS families were uncovered by loss-of-function experiments: RGS1 and RGS13 knockdowns increase chemoattractant signaling in human B lymphoma lines [13], and Rgs1 deletion impairs the entrance of B cells into the lymph nodes and disturbed plasma cell localization in mice [14,15]. However, up to date, function of RGS1 in migration of human plasma cell or plasmacytoma has not been investigated.

In this paper, we investigated the role of RGS1 in human plasmacytoma and plasmablast. We found that augmented expression of RGS1 by lipopolysaccharide (LPS) suppressed the CXCL12-mediated migration and AKT activation in RPMI 8226 plasmacytoma cell line and plasmablasts generated from germinal center B (GC-B) cells. Our findings suggest the important role of RGS1, which regulates the migration via AKT in RPMI 8226 cells and plasmablasts.

## Materials and Methods

### Reagents and antibodies

Recombinant human CXCL12 and IL-21 were purchased from Peprotech (Rocky Hill, NJ). Peptidoglycan (PGN) poly (I:C), LPS, and R848 were purchased from Sigma-Aldrich (Poole, Dorset, UK). Human CpG-B DNA was purchased from Hycult Biotech (Uden, Netherlands). Flagellin was provided by Dr. Myoung Ho Jang (Osaka University). RPMI 1640 medium and fetal bovine serum (FBS) were purchased from Gibco BRL (Eggenstein, Germany). Anti-ERK1/2 and anti-human phospho-AKT1/2/3 monoclonal antibodies (Ab) were obtained from Cell Signaling Technology. Anti-ß-actin and anti-glyceraldehyde-3-phosphate dehydrogenase (GAPDH) Abs were obtained from Santa Cruz (Paso Robles, CA). Anti-RGS1 Ab was purchased from Novus Biologicals (Littleton, CO). The anti-CXC chemokine receptors (CXCR) 4-APC, TCRαβ-FITC, CD38-PerCP-Cy5.5, and CD20-APC were purchased from BD Biosciences (Heidelberg, Germany). The BCA protein reagent was purchased from Thermo Scientific (Hudson, NH). Interleukin (IL)-2 and IL-4 were provided by Prof. Jongseon Choe (College of Medicine, Kangwon National University).

### Cell culture

The human RPMI 8226 cell line was purchased from the American Type Culture Collection (Manassas, VA) and grown in RPMI 1640 media supplemented with 10% (v/v) FBS. The culture was maintained at 37°C in a 5% CO_2_ atmosphere. For treatment with Toll-like receptor (TLR) ligands, 5 × 10^5^ cells were harvested, resuspended in 500 μL fresh media, and stimulated with PGN (10 μg/mL), poly (I:C) (1 μg/mL), LPS (10 ng/mL), flagellin (50 ng/mL), R848 (3 μM), and CpG-B (2 μM) for 18 hours. For stimulation with CXCL12, 5 × 10^5^ cells were harvested, washed once with PBS, and resuspended with 500 μL of 0.5% BSA-DMEM. The cells were subsequently incubated with 100 ng/mL CXCL12 for 5 minutes at 37°C.

### Quantitative RT-PCR

For quantitative RT-PCR, total cellular RNA was prepared from 5 × 10^5^ cells using NucleoSpin RNA II (Macherey-Nagel, Postfach, Duren, Germany). For reverse transcription, cDNA was synthesized from 1 μg isolated total RNA using the iScript cDNA Synthesis Kit (Bio-Rad, Hercules, CA). The gene-specific primers were designed as follows: RGS1, 5’-TTGAGTTCTGGCTGGCTTGTG-3’ (sense) and 5’-GCAGCATCTGAATGCACAAATG-3’ (anti-sense); Blimp-1, 5’-ATCTCAGGGCATGAACAAGG -3’ (sense) and 5’-ATGGGAAGGCTATGCAAACA -3’(anti-sense); Bcl-6, 5’- CTGCAGATGGAGCATGTTGT-3’(sense) and 5'- TCTTCACGAGGAGGCTTGAT -3'(anti-sense); S18, 5’-TTTGCGAGTACTCAACACCAACA-3’ (sense) and 5’-CCTCTTGGTGAGGTCAATGTCTG-3’ (anti-sense). One μL of the cDNA template and 5 μM of the specific primer were used in the Power SYBR-Green PCR kit (Applied Biosystems, Foster City, CA) with a quantitative PCR reaction mixture (20 μL). The following PCR conditions were used in the Step-One Real-Time PCR System (Applied Biosystems, Foster City, CA): 95°C for 10 minutes, 40 cycles at 95°C for 15 seconds, and 60°C for 1 minute. Melting curve analysis was performed to control for the specificity of PCR product fluorescence. Fold induction was calculated using the comparative Ct method [16], and the expression of the ribosomal protein S18 was used as a reference. Data are presented as the means ± standard deviation (SD) of triplicate experiments.

### Western blot analysis

Fifty μg protein was separated using 10% SDS-polyacrylamide gel and electrophoretically transferred to an Immune-Blot PVDF membrane (Bio-Rad, Hercules, CA). The membrane was blocked for 1 hour at RT using TBS/0.1% Tween 20 that contained 5% BSA, and then incubated overnight with the primary Ab. The unbound primary Ab was removed by washing the membrane 3 times with TBS/0.1% Tween 20, followed by incubation with horseradish peroxidase-conjugated anti-rabbit or anti-mouse secondary antibody (diluted 1:3000 in TBS/0.1% Tween 20). The protein was then visualized using enhanced chemiluminescence solution (SuperSignal West Pico Chemiluminescent Substrate; Thermo Scientific, Hudson, NH) and the ImageQuant LAS 4000 biomolecular imager (GE Healthcare Life Sciences, Waukesha, WI). For quantitative analysis of the western blot images, Gel Analyzer software (http://www.gelanalyzer.com) was used. Gel Analyzer allows the measurement of peak height and volume of the band of the expected molecular weight. The background subtraction tools allow subtracting the defined background from the intensity values. Equal loading was confirmed by stripping the western blot and reprobing for GAPDH. The values of the western blot represent relative density of the bands normalized to GAPDH.

### Immunocytochemistry (ICC)

To stain and assess the immunofluorescence of RGS1, 2 × 10^5^ cells were harvested, washed with PBS, and seeded onto glass slides. Cells were fixed with 1% paraformaldehyde and permeabilized with 0.1% Triton X-100. Cells were then incubated for 2 hours with human RGS1 Ab (diluted 1:200). After washing with PBS, the cells were incubated for 1 hour with Cy5-conjugated anti-rabbit secondary antibody (Jackson ImmunoResearch Laboratories, West Grove, PA) and diluted 1:100 in PBS and 4’,6’-diamidino-2’phenylindole (DAPI; Molecular Probes, Invitrogen, Eugene, OR). The specificity of RGS1 antibody used in this study was verified by peptide competition assays. Peptide competition assays was performed by preincubation of anti-RGS1 antibody with purified RGS1 protein (Abnova, Taipei City, Taiwan) on a rotating platform for 20 min at RT before addition of the antibody to the cells. For the competition analysis, the RGS1 antibody was incubated with purified RGS1 protein (Abnova, Taipei City, Taiwan) for 20 minutes before application to the cells. Fluorescence was observed using confocal microscopy (LSM710; Carl Zeiss, Jena, Germany).

Immunostaining of the plasmablasts was carried out using autoimmunostainer BenchMark XT (Ventana Medical Systems, Tucson, AZ, USA) according to the manufacturer’s instructions. In brief, sections of 4μm of agarose-embedded plasmablasts were mounted on silanized charged slides and allowed to dry for 10 min at room temperature and then for 20 min at 65°C. After deparaffinization, heat-induced epitope retrieval using standard Cell Conditioning 1 was performed for 24 min. Subsequently, primary anti-kappa light chain (1:10,000, R10-21-F3, Dako, Glostrup, Denmark) and primary anti-lambda light chain (1:2,000, N10/2, Dako) were labeled using an automated immunostaining system with the OptiView DAB Detection Kit (Ventana Medical Systems, Tucson, AZ, USA). Immunostained sections were counter-stained with hematoxylin.

### Transwell migration assay

To assess chemotactic migration, the plasmablasts and RPMI 8226 cells were harvested and washed once with PBS. After washing, 1 × 10^5^ cells were resuspended in 100 μL 0.5% BSA-DMEM and added to the upper chamber of the transwell membrane (Transwell Permeable Support with a 5.0-μm polycarbonate membrane, 6.5-mm insert, and 24-well plate; Corning Costar, Tewksbury, MA), and 600 μL of 0.5% BSA-DMEM, with or without 100 ng CXCL12, was added to the bottom chamber. After 5 hours of incubation, the top cells (i.e., non-migrated) were removed, and the bottom cells (i.e., migrated) were collected and the number of migrated cells were counted using propidium iodide (PI) exclusion on a BD Accuri C6 flow cytometer as described previously [17]. In detail, the numbers of PI negative cells were counted in 200 μl acquired sample by Accuri C6. Total numbers of migrated cells were obtained by the number of cell in 200μl × 3 (multiplication factor). Data are presented as the means ± SD of triplicate experiments.

### Transfection

RGS1 knockdown with siRNA transfection was performed using a Neon electroporation device (MPK5000, Invitrogen, Carlsbad, CA) according to the manufacturer’s instructions. For each electroporation, 2 × 10^6^ cells were harvested and washed twice in PBS. Then, the cells were resuspended in 100 μL resuspension buffer, transfected with 1 μM of RGS1-targeting siRNA or negative control siRNA at 1200-40-1 (voltage-width-number). Cells were immediately transferred to a 6-well plate containing prewarmed media without antibiotics and incubated at 37°C. One day after transfection, cells were electroporated under the same conditions. RGS1-targeting siRNA was designed as follows: 5’-CUGUAAAGCAGAAGAGAUAUU-3’ (sense), 5’-UAUCUUCUUCUGCUUUACAGUU-3' (anti-sense). Negative control siRNA (nontargeting scrambled RNA [Scr]) was purchased from Genolution Pharmaceuticals (Seoul, South Korea).

### Development of human plasmablasts

This study was approved by the Asan Medical Center Institutional Review Board (Approval number 2013–0864). Informed consents were waived by the Institutional Review Board on the following bases: 1) there was no additional risk to the participants. Remainder tissue samples from routine tonsillectomy and pathologic exam were used for the *in vitro* experiment to study the general phenomena; 2) patient identities were anonymized and completely delinked from unique identifiers. GC-B cells were isolated from human tonsils obtained from remainder tissues of routine diagnostic pathologic examination after therapeutic tonsillectomy. Tonsillar mononuclear cells were isolated by mechanical disruption, which was followed by Ficoll-Paque density gradient centrifugation (GE Healthcare, Waukesha, WI). GC-B cells were purified from tonsillar mononuclear cells using Magnetic-activated cell sorting (Miltenyi Biotec, Auburn, CA), as previously described [18]. Purity was > 95%, as assessed by CD20 and CD38 expression. GC-B cells (2 × 10^5^ cells/well) were cultured in 24-well plates in the presence of irradiated CD154L cells (2 × 10^4^ cells/well [19]), IL-2 (30 U/mL), and IL-4 (50 U/mL) for 4 days. The cultured cells were harvested and counted, and 1 × 10^5^ cells were cultured in the presence of irradiated HS-5 cells, IL-2 (30 U/mL), and IL-21 (30 ng/mL) for 3 days.

### ELISA

Nunc-Immuno MicroWell 96-Well Plates (Thermo Scientific, Marietta, OH, USA) were coated with 10 ug/ml goat anti-human Ig(H+L)-UNLB in PBS overnight at 4°C, washed with PBST (PBS, 0.05% Tween 20) and blocked with 1% BSA for 1 hour. The plates were washed with PBST and incubated with culture soup and serial 5-fold dilutions of Human reference serum from 250 ng/mL in 100uL of PBST for 1 hour. The plates were washed with PBST, incubated with goat anti-human Ig(H+L)-HRP in PBST for 1 hour at RT, washed with PBST, and developed by addition of TMB substrate (PIERCE Rockford, IL, USA). The reaction was stopped with 2% oxalic acid, and the absorbance at 415 nm was measured using a Sunrise microplate reader (TECAN, Männedorf, Switzerland).

### Intracellular staining of phospho-AKT

The intracellular protein staining was performed according to BD phosflow Protocol III. Briefly, after CXCL12 stimulation, the cells were fixed by adding pre-warmed BD Cytofix buffer. For permeabilization, the fixed cells were permeabilized by incubation of chilled BD Perm buffer III for 30 minutes. After permeabilization, the cells were incubated with PE- Mouse anti-AKT (catalogue number 554655) for 1 hour.

### Statistical analysis

All experiments were repeated at least 3 times, unless indicated otherwise in figure legends. The levels of significance for comparison between samples were determined by t-test, using GraphPad Prism Software, version 5 (GraphPad Software, San Diego, CA, USA). The data in the graphs are expressed as the mean ± SD. *P* < 0.05, was considered statistically significant.

## Results

### LPS increases RGS1 expression in RPMI 8226 cells

**Fig 1:**
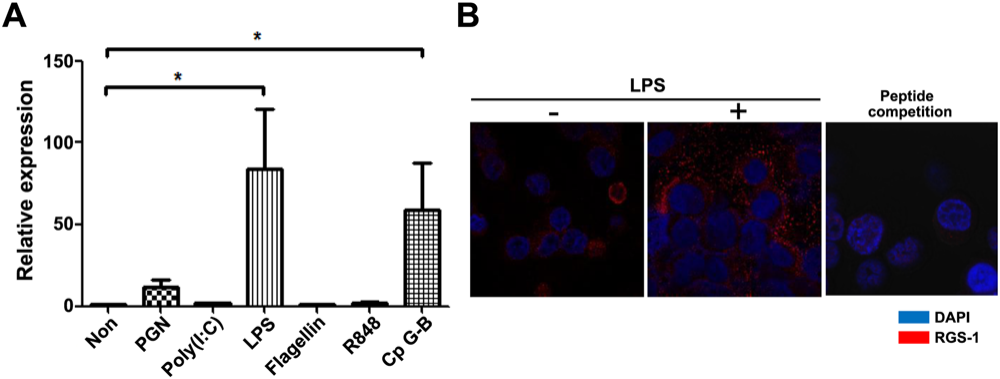
LPS increases RGS1 expression in RPMI 8226 cells. A) Quantitative PCR analysis of RGS1 expression in TLR ligand-treated RPMI 8226 cells. Cultured cells were stimulated with PGN (peptidoglycan) (10 μg/mL; TLR2), poly (I:C) (1 μg/mL; TLR3), LPS (10 ng/mL; TLR4), flagellin (50 ng/mL; TLR5), R848 (3 μM; TLR7), and CpG-B (2 μM; TLR9). After stimulation, cells were harvested and quantitative PCR analysis was performed, as described in the Materials and Methods section. Data show the relative expression levels of RGS1 normalized to the relative S18 expression level of each mRNA (*p <0.05). B) RGS1 expression in LPS-treated RPMI 8226 cells. Cells were cultured in the presence or absence of LPS for 18 hours. To examine the specificity of the RGS1 antibody, the peptide competition assay was performed by addition of RGS1 peptide (1:1 molar ratio with antibody) in antibody binding reaction of ICC. Images shown are the representatives taken under 1000× objective magnification by a confocal microscope.

TLR signaling increases the expression of RGS1 in dendritic cells [20]. We first investigated RGS1 expression in various TLR ligand-treated RPMI 8226 PC cells. RPMI 8226 cells were treated with 10 μg/mL PGN, 10 μg/mL poly (I:C), 10 ng/mL LPS, 50 ng/mL flagellin, 3 μM R848, and 2 μM CpG-B for 18 hours. PGN, LPS, and CpG-B DNA markedly enhanced RGS1 expression according to our quantitative RT-PCR analysis (> 5 fold; Fig 1A). LPS most strongly induced RGS1 expression in comparison with other TLR ligands. We also examined the expression of the RGS1 protein with ICC (Fig 1B). In ICC, LPS noticeably increased RGS1 expression. Negligible RGS1 staining occurred according to the peptide competition analysis, thereby indicating that the Ab specifically binds to cellular RGS1. Overall, these results indicate that LPS strongly induces RGS1 expression.

### LPS reduces CXCL12-mediated chemotaxis in RPMI 8226 cells by inducing RGS1

**Fig 2:**
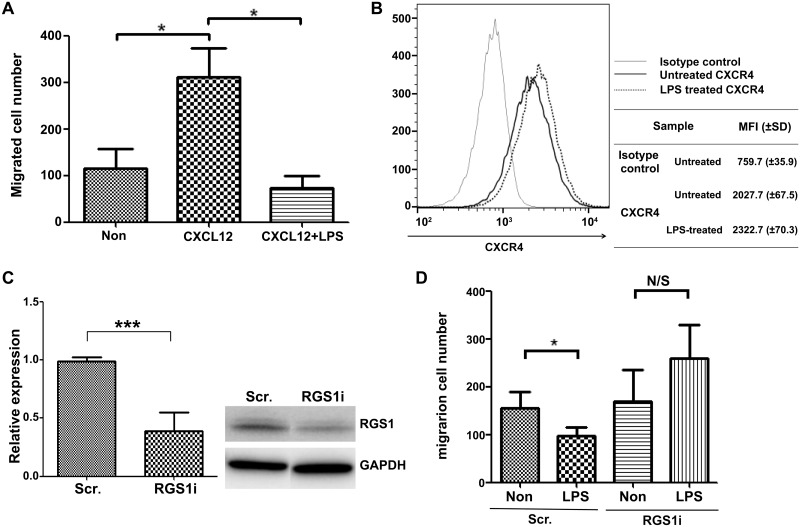
LPS reduces CXCL12-mediated migration by RGS1 in RPMI 8226 cells. RPMI 8226 cells (with or without LPS treatment) were assessed using the transwell migration assay. The upper chamber contained 1 × 10^5^ cells in 100 μL media, and the lower chamber contained 600 μL media. A) LPS reduced CXCL12-mediated transwell migration. Cells were cultured in the presence or absence of LPS for 18 hours before assessment using the migration assay. Cells were allowed to migrate for 5 hours in the absence or presence of 100 ng CXCL12 in the lower chamber of the transwell assay. After 5 hours of migration, 300 μL of the lower chamber media were removed, and cells were counted using FACS and PI. B) CXCR4 expression in LPS-treated RPMI 8226 cells. After 18 hours of incubation with LPS, 2 × 10^5^ cells were harvested, resuspended, and incubated with CXCR4-APC or mouse IgG-APC for 20 minutes on ice. The surface CXCR4 expression was then analyzed using FACS. C) In total, 2 × 10^6^ cells were transfected using a Neon electroporator with siRNA-targeting RGS1 (RGS1i) or nontargeting scrambled RNA (Scr). Three days after electroporation, cells were stimulated with LPS for 18 hours. The reduction in RGS1 RNA expression was assessed using quantitative PCR and RGS1 protein expression was accessed with western blot by anti-RGS1 antibody. D) Transwell migration of Scr-transfected cells and RGS1i-transfected cells, with or without LPS stimulation (*p < 0.05; ***p < 0.0005; N/S: nonsignificant).

RGS proteins reduce CXCL12-induced lymphocyte migration by desensitizing the CXCL12-induced intracellular signaling pathway [6]. We next investigated if LPS reduces CXCL12-induced migration by inducing RGS1 in RPMI 8226 cells. According to the transwell migration assay, CXCL12 induced the migration of RPMI 8226 cells. LPS treatment reduced migration that was induced by CXCL12 by 62% (Fig 2A). To investigate the mechanism of the LPS-mediated reduction in migration, we first measured alterations in the CXCL12 receptor via the expression of CXCR4. Flow cytometry showed that CXCR4 expression on RPMI8226 cells demonstrated no difference between cells, regardless of LPS treatment (Fig 2B). This result suggests that LPS does not affect the surface expression of CXCR4. Instead, LPS was more likely to alter the intracellular signaling pathway. Subsequently, we knocked down RGS1 to examine the role of RGS1 in reducing LPS-induced migration in RPMI 8226 cells, as RGS1 is a known proximal regulator of CXCL12 signaling. In knockdown cells, RGS1 mRNA expression was reduced by 61% and protein expression was reduced significantly as shown in the western blot analysis (Fig 2C). Transwell migration assay with CXCL12 showed that LPS-mediated reduction in migration was abolished by RGS1 knockdown (Fig 2D). These results strongly suggest the important role of RGS1 in LPS-mediated reduction in chemotactic migration by CXCL12 in RPMI 8226 cells.

### CXCL12-induced AKT phosphorylation is reduced by LPS treatment

**Fig 3:**
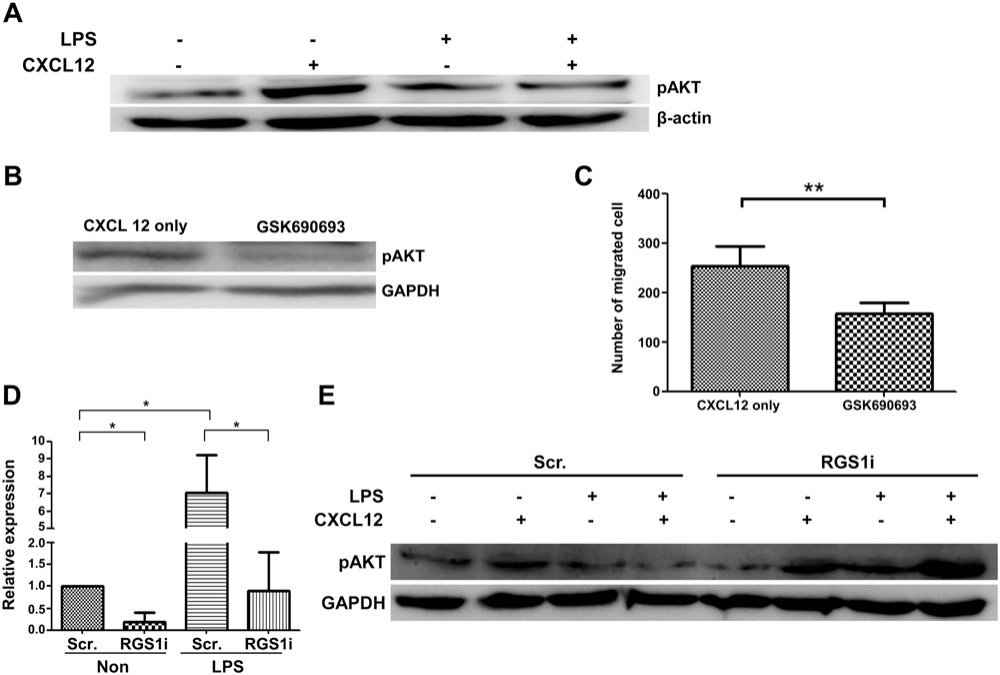
CXCL12-induced migration via AKT is reduced by LPS treatment. A) RPMI 8226 cells were stimulated with 100 ng/mL CXCL12 for 5 minutes in the presence or absence of 10 ng/mL LPS. To determine the decrease in AKT phosphorylation induced by CXCL12 in LPS-treated cells, cells were assessed using western blot analysis for phosphorylated ERK1/2, phosphorylated AKT, and β-actin. B–C) AKT activation is required for the CXCL12-mediated migration of RPMI 8226 cells. Cells were treated with an AKT-specific inhibitor (GSK690693) for 2 hours, and the treated cells were assessed by western blot and CXCL12-mediated transwell migration assays. To knockdown RGS1 in RPMI 8226 cells, the cells were transfected with RGS1i using a Neon electroporator. Three days after electroporation, the cells were stimulated with LPS for 18 hours. D) Reduction in RGS1 expression in RGS1i-transfected RPMI 8226 cells. E) Western blots of ERK and AKT phosphorylation in Scr- and RGS1i-transfected cells. The decrease in AKT phosphorylation induced by CXCL12 in LPS treated-cells was reduced in RGS1i-transfected cells (*p < 0.05; **p < 0.005; ***p < 0.0005).

To define the mechanism that is responsible for CXCL12-induced migration in RPMI 8226 cells, we measured ERK and AKT phosphorylation. ERK and AKT phosphorylation were induced by CXCL12 treatment in RPMI 8226 cells. Interestingly, LPS treatment did not affect ERK phosphorylation by CXCL12 (S1 Fig), but suppressed the CXCL12-induced activation of AKT (Fig 3A). To determine if AKT activation is responsible for the migration of RPMI 8226 cells, we applied specific AKT inhibitors and measured migratory properties using the transwell migration assay. A specific inhibitor of AKT kinase, GSK690693, inhibited AKT phosphorylation (Fig 3B) and attenuated CXCL12-induced migration by 56% (Fig 3C). These results suggest that suppressing AKT activation could be a mechanism for reducing LPS-induced migration in RPMI 8226 cells.

### LPS-induced decreases in AKT phosphorylation are abolished in RGS1-knockdown RPMI 8226 cells

To determine if increases in RGS1 are responsible for suppressing AKT phosphorylation in RPMI 8226 cells, we measured AKT phosphorylation in RGS1-knockdown RPMI 8226 cells. As shown in Fig 3D, RGS1-targeting siRNA reduced the expression of RGS1 by 82% in comparison with Scr-transfected cells. LPS treatment induced RGS1 expression by > 7-fold in comparison with Scr-transfected cells. However, RGS1 induction was noted in RGS1-knockdown cells. This result shows that RGS1 knockdown successfully suppresses the induction of RGS1, even in LPS-treated cells. AKT phosphorylation increased when CXCL12 was used to treat both control and RGS1-knockdown cells. LPS suppressed AKT phosphorylation in control cells, regardless of CXCL12treatment. However, in RGS1-knockdown cells, LPS did not suppress AKT phosphorylation by CXCL12 (Fig 3E). These results suggest that LPS suppresses AKT phosphorylation in RPMI 8226 cells via RGS1.

### LPS also reduced CXCL12-mediated migration in cultured plasmablasts via RGS1-AKT

**Fig 4:**
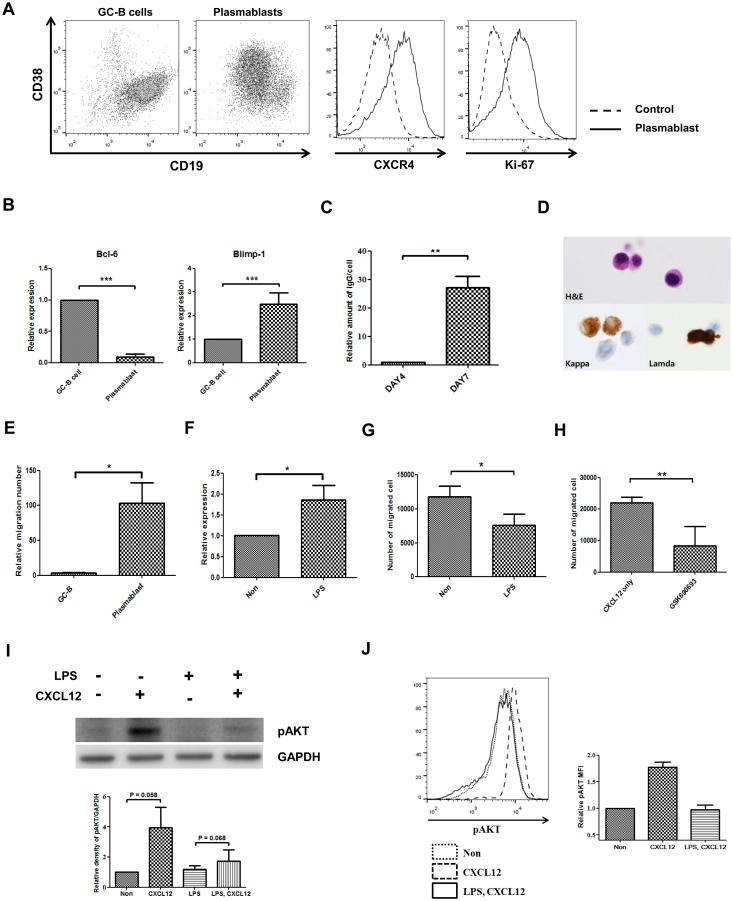
LPS reduces CXCL12-mediated migration via RGS1-AKT in plasmablasts. GC-B cells were cultured in combination with IL-2, IL-4, and IL-21 for 7 days and differentiated into plasmablasts. The cultured plasmablasts were stimulated with 10 ng/mL LPS for 18 hours. RGS1 expression and AKT phosphorylation were assessed in LPS-treated plasmablasts, in addition to assessment using the transwell migration assay. A) CXCR4, CD38, CD19, and Ki-67 expression levels in the cultured plasmablasts. B) Expressions of Bcl-6 and Blimp-1 were measured by real-time PCR at GC-B cells and cultured plasmablast. C) Amount of secreted immunoglobulin was measured at culture day4 and culture day 7. D) After 7 days of culture, purified GC-B cells differentiated to plasmablasts. The cytoplasm is abundant and the nucleus characteristically shows the eccentric position. The nucleus shows fine reticular or mildly blocked chromatin pattern. These plasmablasts show strong cytoplasmic staining for the kappa or the lambda light chain immunocytochemical staining. (H&E, Hematoxylin and eosin stain × 1,000; Kappa, anti-kappa light chain, × 1,000; Lamda, anti-lambda light chain, × 1,000). E) Plasmablasts demonstrate high migratory properties towards CXCL12. F) LPS treatment induces RGS1. G) LPS reduces CXCL12-mediated transwell migration. H) AKT-specific inhibitors reduce migration. I) LPS reduces CXCL12-mediated AKT phosphorylation. Representative western blot of four repetitive experiments is shown. Quantification of phospho-AKT expression was shown in Graph. Phospho-AKT expression was normalized to control levels and corrected for loading differences using GAPDH. J) Intracellular staining of phospho-AKT showed the reduction of the phospho-AKT by LPS treatment. Representative data of two repetitive experiments was shown. Average mean fluorescence intensity (MFI) was shown in graph (*p <0.05; **p <0.005***p <0.0005).

To determine if the LPS-RGS1-AKT axis regulates the migratory properties of human plasmablasts, we generated migrating plasmablasts *in vitro* from tonsillar GC-B cells [21,22]. Purified GC-B cells (> 95%) were cultured with IL-2 and IL-4 for 4 days in the presence of CD154, then with IL-2 and IL-21 without CD154 for 3 days. On day 7, GC-B cells differentiated to CXCR4-, CD38-, and CD19-positive cells (Fig 4A). Proliferation was determined according to Ki-67 expression. We also observed the reduction of Bcl-6, increase of Blimp-1 expression and increased amount of secreted immunoglobulin in culture media compare to the GC-B cells (Fig 4B and 4C). The differentiated cells had typical shape of plasma cells showing basophilic cytoplasm and an eccentric nucleus with heterochromatin. Strong cytoplasmic positivity of immunoglobulin heavy and light chain were seen on ICC (Fig 4D). Furthermore, the differentiated cells demonstrated almost 100-fold more migration towards CXCL12 in comparison with GC-B cells (Fig 4E). These characteristics are consistent with plasmablasts [23,24,25]. LPS treatment also significantly increased RGS1 expression by 186% in cultured plasmablasts (Fig 4F). According to the results of the transwell migration assay with CXCL12, about 10% of input plasmablasts were migrated and LPS reduced CXCL12-induced migration by 40% (Fig 4G). These data show that LPS induces RGS1 expression, and RGS1 may inhibit migration in plasmablasts. Reducing migration using an AKT-specific inhibitor significantly reduced migration to 70% (Fig 4H). Subsequently, we investigated intracellular signaling in plasmablasts. In accordance with the RPMI 8226 cell data, CXCL12 induced AKT phosphorylation and LPS suppressed CXCL12-mediated AKT phosphorylation in plasmablasts (Fig 4I and 4J). These data show that LPS suppresses AKT activation and migration and induces RGS1 expression in plasmablasts, similar to RPMI 8226 cells, suggesting the probable role of RGS1 in CXCL12-induced migration in plasmablasts.

## Discussion

The present study examines the functional role of RGS1 in regulating the CXCL12-mediated migration of RPMI 8226 plasmacytoma cells and plasmablasts. Our results show that RGS1 suppresses AKT activation and regulates CXCL12-mediated migration. RGS1 knockdown abolished the LPS-mediated reduction in AKT activation and migration in RPMI 8226 cells, demonstrating that RGS1 regulates chemotactic migration via AKT activation. These findings are consistent with previous studies, which document that some RGS proteins regulate AKT phosphorylation. RGS4 expression attenuates Sumatriptan-induced AKT activation in BE(2)-C neuroblastoma cells [26]. RGS16 overexpression decreases AKT activation and CXCL12-induced migration, and RGS16 knockdown increases AKT activation and the migration of MO7e megakaryocytes [27]. Similarly, increased RGS13 attenuates AKT activation and migration in HMC-1 mastocytoma cells [28]. Furthermore, RGS3 inhibits AKT phosphorylation by Gβ1γ2 in COS-7 cells [29]. These studies also show that RGS controls migration by regulating ERK activation. According to our results, however, RGS1 does not inhibit ERK activation or CXCL12-induced migration in RPMI 8226 cells (S1 Fig). There is another discrepancy in our data. Splenic B cells from RGS1 knockout mice have significant increase of CXCL12-mediated migration [15]. However, knockdown of RGS1 in RPMI 8226 plasmacytoma cells did not significantly increased the migration (comparison of first and third column in Fig 2D) induced by CXCL12. Interestingly, knockdown of RGS1 in HS-sultan Burkitt's lymphoma cells enhances migration only marginally [13]. Because complex cell signals by RGS proteins affect diverse pathways in various cells, these discrepancies can be explained by cell type-dependent phenomena.

The signaling molecules involved in the migration of myeloma or plasma cells are currently under investigation. CXCL9, CXCL10, and CXCL11 reportedly induce CXCR3-mediated chemotaxis in inflammatory sites [2,30]. CXCL12 signals can be transduced through CXCR4 or CXCR7 in plasma cells. Among these, the CXCL12-CXCR4 axis is crucial for attracting multiple myeloma (MM) and plasma cells to bone marrow [31]. The CXCR4-mediated migration of MM cells to bone marrow is critical for their survival [3]. The CXCR4 inhibitor, AMD3100, interrupts the migration of MM cells to bone marrow stromal cells and sensitizes myeloma cells to overcome drug resistance [4]. Despite the importance of regulating plasma cells and the migration of MM cells, the molecular mechanisms that regulate plasma cell migration remain poorly understood.

We here report for the first time that RGS1 regulates AKT activation during CXCL12-mediated migration. Plasma cells have a high level of RGS1 [15], and subsequently AKT activation is an important part of MM migration [3] and Waldenstrom macroglobulinemia [32]. Thus, regulating AKT activation via RGS1 is an important part of controlling the migration of RPMI 8226 cells and plasmablasts. Further elucidating this signaling pathway in plasma cells would provide novel insights into potential therapeutic modalities for the treatment of plasma cell diseases.

## Supporting Information

S1 Fig ERK activation is not required for CXCL12-mediated migration. RPMI 8226 cells were stimulated with 100 ng/mL CXCL12 for 5 minutes in the presence or absence of 10 ng/mL LPS. Cells were treated with a MEK inhibitor (U0126) for 2 hours, and the treated cells were assessed by western blot and CXCL12-mediated transwell migration assays.
(TIF)
