# Supplementary figures and images for: Regulator of G Protein Signaling 1 Suppresses CXCL12-Mediated Migration and AKT Activation in RPMI 8226 Human Plasmacytoma Cells and Plasmablasts
Source: PLoS One. 2015 Apr 21;10(4):e0124793. doi: 10.1371/journal.pone.0124793 (PMC4405207; doi:10.1371/journal.pone.0124793)

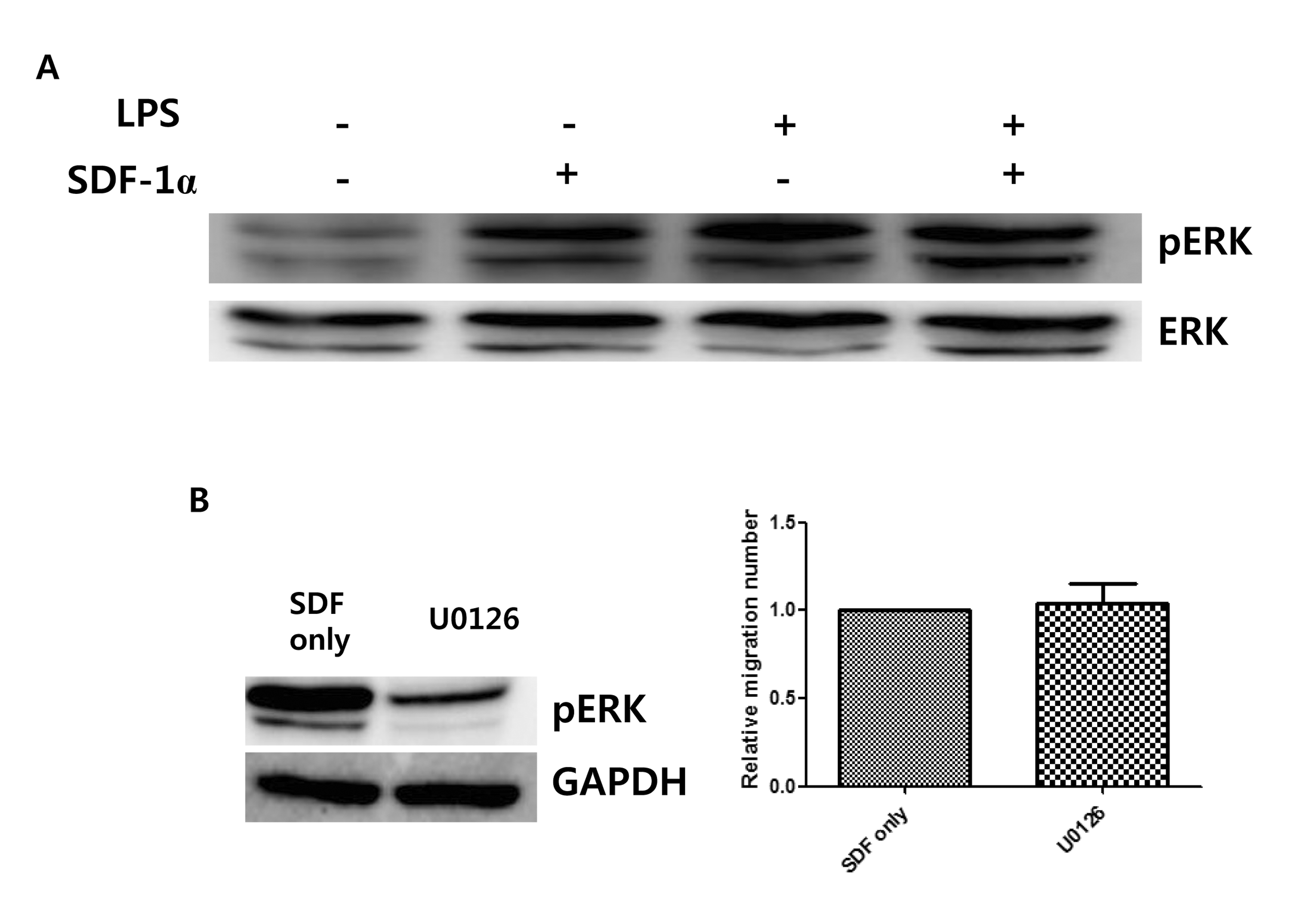

Supplement: S1 Fig — RPMI 8226 cells were stimulated with 100 ng/mL CXCL12 for 5 minutes in the presence or absence of 10 ng/mL LPS. Cells were treated with a MEK inhibitor (U0126) for 2 hours, and the treated cells were assessed by western blot and CXCL12-mediated transwell migration assays. (TIF) [file pone.0124793.s001.tif]
